# “It Was a Downward Spiral”: A Qualitative Study of Young Adult Cancer Survivors’ Experiences with Cognitive and Mental Health

**DOI:** 10.3390/cancers16223819

**Published:** 2024-11-13

**Authors:** Danielle B. Tometich, Christina Hersh, Melinda L. Maconi, Hayden J. Fulton, Dinorah Martinez Tyson, Kellie Zambrano, Syed Hasan, Taylor Welniak, Yvelise Rodriguez, Crystal Bryant, Lisa M. Gudenkauf, Xiaoyin Li, Damon R. Reed, Laura B. Oswald, Andrew Galligan, Brent J. Small, Heather S. L. Jim

**Affiliations:** 1 USF Health, University of South Florida, 4202 E. Fowler Ave, Tampa, FL 33620, USA; 2 Moffitt Cancer Center, 12902 USF Magnolia Drive, Tampa, FL 33612, USA

**Keywords:** young adult cancer survivors, psycho-oncology, mental health, cognitive function, cognitive impairment

## Abstract

**Background/Objectives:** Cancer-related cognitive impairment (CRCI) is common and distressing for young adult (YA) cancer survivors; however, subjective CRCI is often attributed to psychological distress rather than true cognitive change. Thus, we aimed to generate hypotheses regarding potential common biobehavioral mechanisms of CRCI and psychological distress. **Methods:** We conducted semi-structured interviews with YA cancer survivors in peak years for frontal neurocognitive development (ages 18–30), and we asked them to describe their experiences with cognitive and mental health changes during and after treatment for their cancer. An applied thematic analysis was used to identify themes. **Results:** The participants were 20 YA cancer survivors who were, on average, 23 years old at diagnosis and 26 years old when interviewed. Five themes emerged regarding the connection between cognitive and mental health: depressed mood after diagnosis that decreased mental engagement, cancer-related anxiety consumed cognitive resources during treatment, a bidirectional “downward spiral” during treatment, frustration with forgetfulness, and self-criticism about long-term cognitive limitations. Additionally, participants described receiving minimal information about evidence-based strategies for managing CRCI. **Conclusions:** Future research is needed to examine common neurological mechanisms among symptoms of depression, anxiety, and CRCI and to address gaps in survivorship care for YA survivors with CRCI. Cognitive–behavioral interventions may address both cognitive and mental health.

## Simple Summary

About half of young adults (YAs) who have had cancer experience changes in their ability to think, concentrate, and remember; however, these cognitive changes are subtle, difficult to measure, and often dismissed as attributable to anxiety or depression. We suspect that cognitive and mental health changes during and after cancer treatment may be linked by underlying physical and psychological changes. We interviewed 20 YA cancer survivors about their experiences with cognitive and mental health changes during and after cancer treatment. Participants described feeling depression after diagnosis; feeling anxious during treatment, interfering with their ability to think; experiencing cognitive and mental health challenges that exacerbated one another; and feeling frustrated and self-critical about having continued difficulties with cognitive function after treatment. Because YA cancer survivors describe cognitive and mental health as being closely linked, there is potential for psychological interventions to improve both symptoms.

## 1. Introduction

Globally, over one million adolescents and young adults (AYAs; aged 15–39) are diagnosed with cancer each year [1]. Although AYAs represent a minority of those living with cancer, over 85% of them are living for five years or longer after diagnosis, and 23.5 million disability-adjusted life years can be attributed to AYA cancer and treatment sequelae [1,2,3]. As AYA survivors are living long-term after cancer diagnosis and treatment, it is critical to address treatment sequelae with the potential for life-long impacts on their quality of life [4,5,6,7]. One such consequential symptom is cancer-related cognitive impairment (CRCI), or difficulty with attention, concentration, memory recall, or problem-solving. Emerging young adults (YA; ages 18–30) with cancer may be uniquely vulnerable to CRCI, as this age range coincides with a sensitive period of frontal neurodevelopment [8], along with pivotal transition periods for education and career goals [9]. Indeed, up to 53% of YA cancer survivors report cancer-related cognitive impairment (CRCI) that interferes with their work or school [10].

Although cancer survivors report that CRCI is distressing and detrimental to their daily lives, subjective and objective measures of CRCI often have little to no correlation [11,12]. Some experts have argued that subjective or perceived cognitive impairment is a more appropriate measure of CRCI than current gold-standard neuropsychological testing [13], and neuropsychological assessments may not be sensitive enough to detect the impairments experienced and self-reported by cancer survivors [14,15]. In fact, subjective (rather than objective) cognitive impairment is a stronger predictor of worse long-term quality of life in cancer survivors [16]. Despite the recommendation to treat subjective CRCI as a clinically important outcome, research across various age groups attributes subjective CRCI to psychological distress (e.g., depressed mood and anxiety) rather than true cognitive change [17,18,19]. Pre-treatment distress, anxiety, and depression are statistically controlled [20,21,22,23,24] rather than critically evaluating the biobehavioral mechanisms underlying changes to both cognitive and mental health during and after cancer treatment. Meanwhile, findings from research on aging indicate that depression and cognitive impairment may have common neurobiological mechanisms [25,26,27]. This suggests that a closer examination is needed to determine how cancer survivors experience the onset and progression of both CRCI and psychological distress in order to inform future mechanistic and intervention research.

By exploring YA cancer survivors’ lived experience of the connection between their cognitive and mental health during and after cancer treatment, we aimed to generate hypotheses regarding potential biobehavioral mechanisms of both subjective CRCI and psychological distress. Our findings could inform CRCI assessments and interventions to improve both cognitive and mental health in YA cancer survivors.

## 2. Materials and Methods

This qualitative study was conducted at a comprehensive cancer center in the Southeastern United States and approved by the Institutional Review Board (Pro00063210). The methodology used for this study was previously reported in greater detail [28]. Briefly, YA survivors were recruited from an email listserv and clinical database of YA cancer survivors treated at this cancer center. Inclusion criteria were currently age 18–30 years, treated for any non-central nervous system (CNS) cancer in the last five years, no evidence of CNS metastases, able to speak and read English, no unmanaged psychiatric or neurological disorders that could interfere with study participation, no brain injury or stroke within the last five years, and self-report of CRCI by responding yes to both items in a two-item screener (“I have been upset about my problems with memory, concentration, or making mental mistakes” and “I started noticing these problems after my cancer diagnosis or cancer treatment”).

Prior to the interview, participants completed an online survey regarding demographics and were asked to self-report whether they were diagnosed with mental health conditions (i.e., depression, anxiety, and other mental health conditions) and whether each mental health condition was diagnosed before or after their cancer diagnosis.

A semi-structured interview guide addressed experiences of CRCI, factors perceived as contributing to CRCI, and attempted coping strategies. Interviews were guided by the principle of data saturation or continued probing questions until each participant provided their full perspective on their experience with CRCI [29].

Interviewers (KZ and SH) were clinical research staff trained by two PhD-level investigators with experience in qualitative research (DBT and DMT). Participants’ prior contact with interviewers was limited to the informed-consent process. Interviews were completed via HIPAA-compliant videoconference, recorded with participants’ permission, transcribed verbatim, and de-identified. Remuneration in the amount of USD 20 was provided to participants for their time and effort.

An applied thematic analysis of interview transcripts [30] was conducted by qualitative specialists (MM and HF). The inductive analytic approach included developing a preliminary codebook with keywords based on the interview guide and interview transcripts, thorough review of transcripts to identify patterns in quotations containing keywords, and revision of the codebook with emergent codes from quotes with overlapping keywords or where existing keywords did not apply. Intercoder reliability was then evaluated by applying codes to transcript excerpts from at least three interviews during each round of consensus meetings among qualitative coders. Coding discrepancies were discussed during consensus meetings and resolved among qualitative coders and a third author (DBT), when needed. Substantial agreement was reached between raters after the third consensus meeting (Cohen’s kappa = 0.92) and the codebook was finalized [31]. Transcripts were then coded with the finalized codebook using Nvivo 12 (Lumivero, Denver, CO, USA, version 12) [32], and findings were synthesized into themes. Code saturation for applied thematic analysis can be reached with as few as 6 interviews with full thematic saturation at 9 interviews; thus, we oversampled by interviewing 20 participants to ensure a rich understanding of themes [33].

## 3. Results

There were 37 YA cancer survivors approached via telephone by study staff from October 2022 to February 2023, and 20 consented to participate in semi-structured interviews (14 were unable to contact, and 3 refused citing lack of time or interest). On average, participants were 25.5 years old at the time of the study interview and 22.9 years old at cancer diagnosis. The participant characteristics were previously published [28], and overall sample characteristics are shown in Table 1. Regarding diagnosed mental health conditions, 55% reported depression (35% diagnosed before and 20% after cancer), 65% reported anxiety (50% diagnosed before and 15% after cancer), and 20% reported another mental health condition (15% diagnosed before and 5% after cancer).

Interviews were from 17 to 57 min in length, and there were five themes identified related to the connection between cognitive and mental health during and after cancer treatment: depressed mood after diagnosis that decreased mental engagement, cancer-related anxiety consumed cognitive resources during treatment, a bidirectional “downward spiral” during treatment, frustration with forgetfulness, and self-criticism about long-term cognitive limitations after treatment. Additionally, participants described a survivorship care gap such that they were not provided with information about evidence-based strategies for managing CRCI. Themes and abbreviated illustrative quotes are described below (see Table 2 for quotations with greater detail).

### 3.1. Depressed Mood After Diagnosis That Decreased Mental Engagement

Reflecting on the period of time immediately after diagnosis, participants described depressed mood that decreased mental engagement. For example, one participant described how his mood and anhedonia after diagnosis interfered with his motivation for working in his established career as a teacher, stating,

*“It became harder to do lessons and I think it was a little bit of depression as well, like I didn’t want to do anything. I didn’t want to go into work, I didn’t want to teach, I didn’t want to study the lessons”.* (Participant 6, 25–30-year-old male, thyroid cancer, >1 year post-surgery and radiation, no mental health conditions).

Another participant described being “on autopilot” and stated,

*“I repressed a couple things … that I had to deal with mentally afterwards. A lot of the feelings … that I didn’t allow myself to feel I had to acknowledge that once it was over”.* (Participant 10, 25–30-year-old female, Hodgkin’s lymphoma, >6 months post-chemotherapy, depression onset before cancer diagnosis).

### 3.2. Cancer-Related Anxiety/Consumed Cognitive Resources During Treatment

During treatment, participants recalled being easily distracted by feelings of stress, fear, and experiencing cancer as traumatic. In fact, multiple participants used the word “consumed” (Participants 4 and 7) to describe how anxiety about cancer occupied their thoughts, leaving little room for focused attention on present tasks. As one participant stated,

*“My brain has been rewired in a way to kinda focus on other things, more survivalist things I guess”.* (Participant 8, 25–30-year-old female, Hodgkin’s lymphoma, >1 year post-bone marrow transplant, anxiety onset before cancer diagnosis).

Another participant articulated how stress contributes to distraction.

*“I feel like some of the cognitive challenges I have could be related to just being distracted with this stress I have in my life”.* (Participant 13, 20–25-year-old female, thyroid cancer, >2 years post-surgery only, depression and anxiety onset before cancer diagnosis).

### 3.3. A Bidirectional “Downward Spiral” During Treatment

In addition to the effect of anxiety on cognitive function, participants described how cognitive and mental health have bidirectional effects. Although participants acknowledged that these bidirectional effects could be positive or negative, it required effort to create the momentum for positive effects. One participant described how her stress and cognitive function went “hand to hand” in “a downward spiral”, and she has needed to use her years since treatment “just working [her] way back up, trying to get them both to a much better place” (Participant 15, 25–30-year-old female, Hodgkin’s lymphoma, >2 years post-chemotherapy, depression and anxiety onset before cancer diagnosis).

Another participant who completed treatment more recently noted the close relationship between psychoneurological symptoms (fatigue and memory) and low mood.

*“When you have four bad days in a row where you can’t remember things it’s really hard to just continue to wake up with a positive attitude and positive energy, but if you can I think it helps, it’s just it’s really hard to get there because sometimes you have four tired days and there is just nothing you can do about it…”* (Participant 7, 25–30-year-old female, Hodgkin’s lymphoma, <6 months post-chemoradiation, anxiety onset before cancer diagnosis).

### 3.4. Frustration with Forgetfulness

While reflecting on how cognitive impairment results in low mood, participants described feeling frustrated with their forgetfulness. In fact, a participant described frustration as the “main emotion” when she “can’t remember something I’m trying to, or [she] can’t find a word” (Participant 5, 25–30 years old, rare hematologic cancer, 1 month post-immunotherapy, depression onset after cancer diagnosis, and anxiety onset before cancer).

### 3.5. Self-Criticism About Long-Term Cognitive Limitations

Several participants elaborated on the self-critical thoughts that accompanied frustration with their cognitive problems. Multiple participants stated that their long-term cognitive problems following cancer treatment made them feel “stupid” in school environments (Participants 18 and 13) or fearful that their limitations may be permanent or could worsen over time (Participants 7, 11, and 13). As one participant stated,

*“So, emotionally it’s more frustration, and then also fear, right, because you read the horror stories of the people who, their memory didn’t come back … three years down the road and they still experience moments of brain fog, and as a [YA] that’s not what I want … I’m definitely not at the age where that should be a normal part of my life”.* (Participant 7, 25–30-year-old female, Hodgkin’s lymphoma, <6 months post-chemoradiation, anxiety onset before cancer diagnosis).

### 3.6. Survivorship Care Gap for Managing CRCI

A few participants volunteered the perception of a gap in survivorship care for managing CRCI. In particular, they stated that they wanted to receive more information or resources from their oncology care team about the etiology and management of CRCI. One participant described searching for information on her own saying,

*“…if there is anything that has proven to be positive or helpful, I would love to know because I am just stuck with [searching on the internet] and trying to exercise my brain to the best of my abilities”.* (Participant 7, 25–30-year-old female, Hodgkin’s lymphoma, <6 months post-chemoradiation, anxiety onset before cancer diagnosis).

## 4. Discussion

The purpose of this study was to describe YA cancer survivors’ lived experience of the connection between their cognitive and mental health during and after cancer treatment. In doing so, we generated several hypotheses regarding potential common biobehavioral mechanisms of subjective CRCI and psychological distress. Findings are further described below, along with hypotheses and potential avenues for future research.

Participants described experiencing depressive symptoms after diagnosis, along with feelings of amotivation and disconnection. We hypothesize that these depressive and cognitive symptoms after diagnosis but prior to treatment are due to both physiological and psychological mechanisms. Even prior to treatment, AYA cancer survivors have shown objective cognitive impairments associated with elevated proinflammatory cytokines, and less perceived cognitive impairments than healthy controls but a greater perceived impact of their impairments on their quality of life [34]. Similarly, a study of breast cancer patients before surgery and before any systemic treatment found higher rates of objective and subjective cognitive impairment compared to healthy controls, with greater fatigue associated with subjective impairment [35]. Cancer-related physiological changes to inflammation, hormones, and proteins have been linked to pre-treatment psychoneurological symptoms, including cognitive impairment and fatigue [36,37,38]. Additionally, depressed mood and emotional numbing may be symptoms of post-traumatic stress, which has been found to be highly prevalent in YA cancer survivors [39]. Post-traumatic stress symptoms have been found to explain lower cognitive performance in breast cancer survivors [40]. However, research to date has not examined the contributions of both physiological and post-traumatic stress symptoms on pre-treatment CRCI.

Participants highlighted how anxiety consumes cognitive resources, leaving little room for focused attention on present tasks. This is consistent with the hypothesis that anxiety increases distraction from present tasks and stimuli, and this inattention leads to reduced encoding into memory. A meta-analysis has shown that anxiety, whether self-reported or experimentally induced, impairs working-memory performance by approximately one-third standard deviation—a small-to-moderate effect size [41]. Cancer is a substantial source of anxiety, and YA cancer survivors are at increased risk for anxiety disorders compared to YAs without cancer and older cancer survivors, with one in three YA cancer survivors experiencing psychological distress or anxiety [42]. Anxiety has also been associated with both subjective and objective CRCI [43]. Furthermore, animal models have demonstrated that allostatic load from chronic stress leads to neuronal atrophy in the prefrontal cortex and hippocampus, along with neuronal hypertrophy in the amygdala and orbitofrontal cortex [44]. These neurological changes lead to increased sensitivity to anxiety and could be a biological mechanism underlying both anxiety and CRCI [45].

A bidirectional relationship between cognitive and mental health during treatment was described as having a downward or negative impact, and YAs described the effortful process of improving both after treatment. Furthermore, participants reported frustration as the primary emotion experienced in response to cognitive struggles after treatment, and some participants expanded on the frustration with self-critical thoughts and self-directed anger during moments of CRCI. Indeed, feelings of frustration are understandable in response to CRCI; however, intense emotions and difficulty with emotion regulation are also closely linked to executive cognitive functioning [46]. Thus, CRCI and psychological distress may not only exacerbate one another; they also may have common neurological mechanisms, resulting in impaired executive function and emotion regulation. An intervention that includes components of cognitive rehabilitation and emotion-regulation skills, such as Memory and Attention Adaptation Training (MAAT) [47], has strong potential to address these distressing symptoms and promote neuroplasticity. The evidence is growing for the efficacy of MAAT and other cognitive–behavioral approaches [48,49,50], and further research is needed to test the effects for YA cancer survivors and investigate biobehavioral mechanisms of intervention effects.

Although not explicitly discussed in interviews, disruptions to sleep quantity and quality during and after cancer treatment likely have an important role in both cognitive and mental health. One participant mentioned the adverse impact of fatigue on their memory and mood. Furthermore, sleep problems were a noted risk factor for cognitive impairments in our prior report [28]. Sleep problems are pervasive among cancer survivors, with an estimated 40–80% of cancer survivors reporting insomnia during treatment [51,52]. Stress and anxiety due to a cancer diagnosis, fears of recurrence, and side-effects from cancer treatment are all plausible precipitating factors for new or worsening sleep problems [53,54]. Disordered sleep is connected to a plethora of neurobiological changes also associated with cognitive and mental health problems, including HPA-axis dysfunction, dysregulation of serotonin and dopamine systems, and insufficient glymphatic function [55,56,57]. Evidence-based cognitive–behavioral therapy for insomnia (CBT-I) has shown remarkable effectiveness among cancer survivors [58,59,60]. Additionally, CBT-I has shown small-to-moderate effects for improving additional psychoneurological symptoms, such as fatigue, anxiety, depression, and perceived cognitive impairment [60,61,62]. Efforts to increase referrals and improve access to CBT-I among YA cancer survivors would likely be highly beneficial for improving psychoneurological symptoms and quality of life during cancer survivorship.

Finally, participants volunteered their perception of a gap in survivorship care regarding assessment and intervention for their CRCI. A multidisciplinary group of AYA cancer stakeholders has identified survivorship care priorities, including screening for CRCI and referral for neurocognitive, psychosocial, and mental health intervention [63]. This suggests a need for wider dissemination of results from CRCI research and implementation work to enhance connections with informational and intervention services in the clinical setting.

Findings should be considered within the context of our study’s strengths and limitations. Our qualitative research design was appropriate for hypothesis generation rather than testing a priori hypotheses. The present report was supplemental to primary outcomes of this qualitative study that have been previously published [28]; however, a secondary paper was necessary to fully present and discuss findings. The results may not be widely generalizable, as purposive sampling focused on YA cancer survivors reporting CRCI, and we included a volunteer sample recruited from one comprehensive cancer center. One of the strengths of our study was including a diverse sample with respect to cancer types and treatments; however, we were not able to stratify results and examine differences by cancer type or treatment due to the smaller sample size when parsed based on diagnosis or treatment. Future research may consider using online recruitment methods to reach a more nationally representative sample and could consider employing quantitative or mixed-method designs to test hypotheses regarding the biobehavioral mechanisms involved in both cognitive and mental health for YA cancer survivors.

## 5. Conclusions

We conducted a qualitative study to describe the connection between cognitive and mental health among YA cancer survivors. Participants described depressive symptoms after diagnosis but prior to treatment, anxiety during treatment that consumed cognitive resources, and a bidirectional “downward spiral” between cognitive and mental health over time. Notably, feelings of frustration in response to moments of CRCI were common, and some participants also described self-critical thoughts and self-directed anger when experiencing problems with cognitive function. There are opportunities for future research to examine the contribution of both physiological mechanisms (e.g., inflammation) and post-traumatic stress symptoms to the development of pre-treatment CRCI. Additionally, neurological changes due to stress may contribute to both anxiety and CRCI during treatment. Further research is also needed to examine the relationship between emotion regulation and CRCI after treatment and investigate biobehavioral mechanisms of cognitive–behavioral interventions.

## Figures and Tables

**Table 1:** Sample Characteristics (N = 20).

Characteristic	n (%)
Age at interview	
20–25	8 (40.0)
25–30	12 (60.0)
Gender	
Female	15 (75.0)
Male	4 (20.0)
Non-binary	1 (5.0)
Cancer type	
Hodgkin’s lymphoma	4 (20.0)
Leukemia	3 (15.0)
Thyroid	3 (15.0)
Breast	2 (10.0)
Colorectal	1 (5.0)
Bladder	1 (5.0)
Testicular	1 (5.0)
Sarcoma	1 (5.0)
Head and neck	1 (5.0)
Non-Hodgkin’s lymphoma	1 (5.0)
Other hematologic	1 (5.0)
Multiple cancers	1 (5.0)
Most recent treatment	
Chemotherapy	6 (30.0)
Bone marrow transplant	3 (15.0)
Chemoradiation	2 (10.0)
Hormone therapy	2 (10.0)
Immunotherapy	2 (10.0)
Surgery and radiation	2 (10.0)
Surgery only	2 (10.0)
Mental health conditions ^1^	
Depression	11 (55.0)
Anxiety	13 (65.0)
Other mental health condition	4 (20.0)
No mental health condition	6 (30.0)
Mental health condition onset ^2^	
Before cancer	11 (55.0)
After cancer diagnosis	5 (25.0)

^1^ Frequency does not add to 100% due to comorbid mental health conditions. ^2^ Some participants had separate mental health conditions diagnosed before and after cancer diagnosis.

**Table 2:** Themes and illustrative quotes of cognitive and mental health in young adult cancer survivors.

Theme	Illustrative Quotes
Depressed mood after diagnosis that decreased mental engagement	Participant 6: *Probably around the time where I was officially diagnosed is when it started to hit me harder because I knew I had cancer at that time. So, that’s when I started getting–so, I was a teacher for eight years and I’ve done it for many years and I felt like I did really well at it. But just during this time after I got diagnosed, it became harder. It became harder to do lessons and I think it was a little bit of depression as well, like I didn’t want to do anything. I didn’t want to go into work, I didn’t want to teach, I didn’t want to study the lessons.* Participant 10: *I was maybe on autopilot for that time period [immediately after diagnosis]. And I feel like, like I said, I repressed a couple things while dealing with that during that time period that I had to deal with mentally afterwards. A lot of the feelings that I felt maybe during that time period that I didn’t allow myself to feel I had to acknowledge that once it was over.*
Cancer-related anxiety consumes cognitive resources during treatment	Participant 8: *I don’t know if [the cause of memory changes] was just like the high-dose chemo. I’m sure it’s also just like the stress of everything. My brain has been rewired in a way to kinda focus on other things, more survivalist things I guess.* Participant 4: *I think that the biggest thing [that affected memory and concentration]–I think it would–I would say it was pretty much the same except for the fact that I would find myself thinking about it when I wasn’t supposed to like doing work or whatever. I would randomly start thinking of it and be like, “Why am I thinking about that right now?” So, I’d kind of get sidetracked thinking about the diagnosis or the experience…The first word that came to mind [about causes of thinking, memory, and concentration changes] was trauma. I mean, I think it’s a pretty traumatic experience for somebody to go through, and you don’t necessarily realize that in the moment. It’s kinda one of those things that being a little further removed from it, you’re like, “Oh, yeah. That was kind of a pretty crazy wild thing that I went through”. So, I would say just having something that came so suddenly and unexpected in your life to just kind of like dive into your thoughts and consumed them a little bit.* Participant 7: *Oh, I think it [mental health] does impact the brain fog. I don’t think it’s totally responsible for the brain fog but when your mind is completely consumed with just general fear and you do get some dark thoughts and you worry about the worst happening and I did have a decent amount of complications, it kinda muddles all the positive thoughts and all your clear thinking, right. So, your body just kinda changes and it thinks about rather scary things and then you just don’t want to be alone with your thoughts.* Participant 13: *I think it’s been challenging, especially with the anxiety because it’s so easy to focus on the things that are causing me stress. And then I forget to pay attention to other things going on in my life. And so, I feel like some of the cognitive challenges I have could be related to just being distracted with this stress I have in my life.*
A bidirectional “downward spiral” during treatment	Participant 15: *And the more I thought about that [cancer], the more stressed I got and the more frustrated I got. And so, I think that definitely played a role. I really think they went hand-in-hand. So, it was like whether the mental health was affecting the cognition. The cognition is affecting the mental health. I think they really affected each other and it was a downward spiral. And then over really the past few years, I’ve been just working my way back up, trying to get them both to a much better place.* Participant 7: *Oh I definitely think your mood impacts your memory a bit but it’s so hard to completely stay positive all the time when you’re constantly getting frustrated with yourself and you kind of spiral into crankiness and irritability…because you’re not yourself…When you have four bad days in a row where you can’t remember things it’s really hard to just continue to wake up with a positive attitude and positive energy, but if you can I think it helps, it’s just it’s really hard to get there because sometimes you have four tired days and there is just nothing you can do about it and you’re just going to be cranky that you can’t remember things.* Participant 10: *If I kept trying to press myself to remember, it would kinda spiral me into not remembering even more, so that’s why I said I would just kinda be more patient and just take a step back.*
Frustration with forgetfulness	Participant 5: *Frustrated I would say is the main emotion. I get very frustrated when I can’t remember something I’m trying to, or I can’t find a word that I’m trying to use, or like I said, the short-term stuff like, “Did I just wash my hands?” I would say that’s the main thing, and I feel like it’s mainly because I know that there is a difference now than prior to treatment. It’s not the occasional spacey moment, it’s just now I’m unsure.* Participant 14: *It was really very depressing because these were people I loved and cared about and that, and it was the worst feeling ever to tell someone you’re dating, like what’s your name again. I mean that’s the worst thing you can imagine. Part of it, I knew it would happen so it wasn’t too bad of a shock, but again it’s kind of the worst thing ever being like, hey, what’s your name again even though we’ve been friends for eight to nine years or, hey, can we slow it down on the reading or telling my professor I just can’t manage the classes anymore because even though they were basic college classes, I couldn’t even keep up with just reading, it took so much time. So, it was very degrading in a sense and just kind of–I don’t know how to–just degrading, I would say.* Participant 19: *It’s very frustrating. It definitely makes me feel I guess kind of self-conscious and definitely brings in some self-doubt because I am going to medical school in the summer… So, I’m very anxious and stressed about, “Okay, am I going to be able to do it?” Just because I know it’s a lot. So, I do worry about that. Not that I can’t do it, but how hard is it going to be for me to do it compared to other people? Am I gonna struggle more? Am I gonna have to take extra hours to study compared to everybody else? That kind of a thing. Is the workload going to be too much in too short of a time frame? That kind of thing.* Participant 20: *I get very easily frustrated now because I feel like everything I do has to count, otherwise it’s like I’m wasting time. So, it’s a lot of pressure, I think, on myself. Like if I make little mistakes, I get mad. Like, I’ll be playing a video game sometimes and I’ll make some stupid mistake, I’ll put something in the wrong place, and I have to start something over. And I get really angry, and I can’t really come to terms with why, and I feel like it’s always because it goes back to everything that I do has to mean something. And if I make a mistake, I’m taking away from that meaning.*
Self-criticism about long-term cognitive limitations	Participant 18: *Well, sometimes it makes me sad. Sometimes it doesn’t bother me. I’ll tell you how it really affected me was when I figured out it was really, really hard for me to retain I wanna say academic knowledge, that part really has an impact because it makes me feel stupid, you know? So, I would say that is the biggest impact.* Participant 7: *I get very frustrated. I am a perfectionist and I know when I’m forgetting something and I know when my memory is not what I want it to be or what it needs to be. So, I get very frustrated and I’m typically pretty hard on myself, and I’m like, dammit, why can’t I remember this, why can’t remember that? This person just said that to me. So, emotionally it’s more frustration, and then also fear, right, because you read the horror stories of the people who, their memory didn’t quite come back after six months. It’s three years down the road and they still experience moments of brain fog, and as a 28-year-old that’s not what I want. I mean I don’t think it’s what anybody wants but I’m definitely not at the age where that should be a normal part of my life.* Participant 11: *It’s [memory loss] very distressing for me because I feel like I’m insane. I’m worried that I’m losing pieces of my life to the void, basically. It’s scary because if I can’t remember things, is this going to worsen until I can no longer experience my own life? It’s embarrassing as well because I feel helpless, I have to rely on others just to know what’s happened to me. It makes me feel very vulnerable, like someone has to tell me what did or didn’t happen, and I just have to trust that. I have to rely on that person instead of relying on my own mind, so it’s very distressing.* Participant 13: *It definitely makes me a little discouraged at times, especially because I am such a high achiever in school. It can be kind of difficult to process that feeling. My first couple of months, I really had to convince myself I was not stupid because I just felt so–it felt so different than I was before my surgery. And I felt like I had this significant cognitive decline. And I was so nervous that something got messed up permanently.*
Survivorship care gap for managing CRCI	Participant 7: *It would be super helpful if there is anything that has been proven to help with brain fog or kind of trying to break through that if maybe some of the nurse practitioners and hematologists and oncologists could share that a little bit more when we bring up brain fog because they kinda just mark it down and say it’ll go away eventually. But if there is anything that has proven to be positive or helpful I would love to know because I am just stuck with Google searches and trying to exercise my brain to the best of my abilities.* Participant 17: *I would say just how important it is to have resources available for people for different cognitive needs. I think it’s like person by person, like on a case-by-case basis what they need to know.* Participant 19: *Honestly, they’ve never really talked to me about it a whole lot. Just kind of there’s not a lot of research out there on it. … And they always told me, like, “Oh, chemo brain is definitely a thing. We know that”. But I don’t think it was a super -- it’s one of those things where they don’t really know per se how to help you with that because there’s just not a lot of literature on it, that kind of thing. So, they were aware of it, but I don’t think there’s been a lot of discussion with me about how to make it better, I guess… I feel like it really comes across as, like, “Well, not much we can do about it”. It’s like you’re kind of just left with it, like, “Okay”. Because you do have to -- if you’re a young adult, you’re not in your career already, you’re not necessarily done with working towards a goal, so you have to still deal with those changes, and find a what through them. So, maybe nice to have some support with that.*

## Data Availability

The datasets generated and analyzed during the current study are available from the corresponding author upon reasonable request.
